# Modulating Blood-Brain Barrier Metabolites of Broiler Chickens Through Dietary Flaxseed Oil

**DOI:** 10.3390/biom16050661

**Published:** 2026-04-29

**Authors:** Safiu A. Suberu, Paul C. Omaliko, Deji A. Ekunseitan, Nathanael I. Lichti, Bruce R. Cooper, Yewande O. Fasina

**Affiliations:** 1 Department of Animal Sciences, North Carolina Agricultural and Technical State University, Greensboro, NC 27411, USA; sasuberu@aggies.ncat.edu (S.A.S.); pcomaliko@aggies.ncat.edu (P.C.O.); daekunseitan@ncat.edu (D.A.E.); 2 Bindley Bioscience Center, Purdue University, West Lafayette, IN 47907, USA; nlichti@purdue.edu (N.I.L.); brcooper@purdue.edu (B.R.C.)

**Keywords:** hypothalamus, dorsal raphe nucleus, plasma, metabolites, dietary fat

## Abstract

Dietary polyunsaturated fatty acids (PUFAs) are vital for brain health and cognitive function. The dorsal raphe nucleus (DRN) regulates mood via serotonin, while the hypothalamus (HYP) controls energy homeostasis. Flaxseed oil (FLAX) is rich in omega-3 PUFAs like α-linolenic acid (ALA), and has been reported to influence serotonergic signaling in mammals, but data in poultry are scarce. This study investigated the effects of FLAX on metabolites crossing the blood-brain barrier (BBB) to serotonergic brain regions and on growth performance in broiler chickens. Day-old chicks (*n* = 160) were assigned to two diets (5 replicates/treatment): control (CON; poultry fat-based diet) or FLAX (3% inclusion level). Growth performance was recorded, and DRN, HYP, and plasma were analyzed using HPLC-MS metabolomics. Serotonin and its metabolite 5-HIAA were quantified using LC-MS/MS. FLAX-fed birds had higher body weight gain (*p* < 0.0055) and better feed conversion ratio (*p* < 0.0049) than CON. Metabolomics identified 2271 features, of which 650 were annotated as metabolites. Of 35 differentially abundant plasma metabolites, eight were also differentially abundant in brain tissues. In the DRN, tryptophan (serotonin precursor) and corydaline (neuroprotective) were upregulated. Serotonin levels were significantly higher in both the DRN and HYP of FLAX-fed birds compared to CON. This suggest that dietary flaxseed oil may modulate stress responses, behavior, and welfare in broilers. In the HYP, dethiobiotin (energy), galanthamine (neuroprotective), and gambogic acid (antioxidative) were upregulated, while xanthoxyletin (anti-inflammatory) was downregulated. In conclusion, flaxseed oil improved growth and elevated serotonin in the DRN and HYP via enhanced tryptophan availability, suggesting potential benefits for stress resilience and welfare.

## 1. Introduction

Dietary fat plays a significant role in modulating brain metabolite profiles and has different effects on brain function, depending on the type and composition of the fat [1,2,3]. Dietary fats differ based on fatty acid composition, chemical structure, and degree of saturation. They are grouped into saturated fats (SFA) and unsaturated fats. Unsaturated fats are further classified into monounsaturated fatty acids (MUFAs) and polyunsaturated fatty acids (PUFAs). It has been reported that saturated fatty acids are less digestible than unsaturated fatty acids [4]. The composition of fats plays a vital role in modulating the health of broiler chickens, and it has been reported that PUFA and MUFA fat types confer more beneficial qualities compared to saturated fats that tend to increase the risk of diseases, including inflammation, stress, and poor gut health [5].

Flaxseed oil is rich in PUFAs such as Alpha Linolenic Acid (ALA), an ω-3 PUFA. At 40–60%, ALA concentrations in flaxseed oil are considerably higher than in other vegetable sources, such as canola (∼6–8%) [6]. ALA is converted to its long-chain counterparts, eicosapentaenoic acid (EPA) and docosahexaenoic acid (DHA), which are bioactive compounds with established physiological benefits [6,7,8], through elongation and desaturation reactions. The poultry digestive system contains hepatic elongase and desaturase enzymes that trigger the conversion of ALA to its derivatives [6,9]. These PUFAs, EPA and DHA, have been reported to play a crucial role in the function of a chicken’s brain [10,11].

Dietary flaxseed oil provides a plant-based source of omega-3 precursors that must be absorbed, metabolized, and transported to the brain to exert effects. ALA is efficiently absorbed in the small intestine, particularly when emulsified, and transported to the liver [12]. In the liver, ALA undergoes limited elongation and desaturation to EPA and DHA via hepatic elongase and desaturase enzymes [13]. To reach the brain, these omega-3 fatty acids must cross the blood-brain barrier (BBB), a process primarily mediated by the transporter Major Facilitator Superfamily Domain-containing protein 2A (MFSD2A), which selectively imports lysophosphatidylcholine (LPC) forms of DHA and EPA [14]. ALA itself may also cross the BBB via passive diffusion, though this pathway is less characterized [15]. Once inside the central nervous system, these fatty acids and their derivatives can influence neurotransmitter pathways, neuroinflammation, and energy metabolism.

A systematic study of brain metabolites can be used to understand the potential role of flaxseed oil in modulating cognitive function and improving broiler brain health [10,11]. It is reported that brain metabolites are influenced by dietary fats, especially in the brain regions involved in fear responses [16,17,18]. The composition of brain lipids, neuroinflammation, gut microbiota, and brain signaling pathways are influenced by the specific type of dietary fat consumed, consequently impacting the overall profile of brain metabolites [3,18,19]. The Dorsal raphe nucleus (DRN) in chickens is a significant part of the serotonergic system and regulates multiple physiological functions and behaviors, including cognitive processes, neuroendocrine functions, and synaptic plasticity [20,21]. Dietary fats can influence the cells that release neurotransmitters, which can, in turn, influence physiology and metabolic functions. Lizarbe et al. [22] reported that the amount and composition of fat consumed are linked to impaired functions and brain modifications, thus demonstrating the influence of dietary fats on brain chemistry. In the DRN, neurotransmission encompasses a multifaceted interplay of processes involving synthesis, release, activation of receptors, reuptake, and degradation back into the neural cells.

Like the DRN, the hypothalamus plays an underlying role in maintaining primary metabolic processes in poultry birds [23,24]. It controls body temperature, hunger, sleep, growth, and circadian rhythms by stimulating the pituitary, which secretes essential hormones controlling other glands/organs [25]. The hypothalamus releases somatoliberin (GHRH), which induces pituitary somatotropin (GH) secretion, the most crucial growth factor. The hypothalamus functions as a central processor influenced by both external environmental stimuli (e.g., temperature) and internal physiological signals (e.g., hormones and metabolite levels), integrates these cues, and modulates physiological processes [26].

Although dietary fats are known to influence brain serotonergic signaling and behavioral traits in mice and humans, Information about this is scarce in poultry and could have significant implications for bird behavior and poultry welfare. Therefore, this study aims to investigate the influence of flaxseed oil on metabolite composition in the serotonergic brain regions, namely the DRN and HYP, and on broiler growth performance.

## 2. Materials and Methods

### 2.1. Experimental Design, Diet, and Bird Management

All animal procedures were approved by the Institutional Animal Care and Use Committee of North Carolina Agricultural and Technical State University (IACUC Protocol No. 20-004.0). In a 3-week experiment, day-old Ross 708 broiler male chicks (160) were commercially sourced and housed at the Poultry Research Unit of the North Carolina Agricultural and Technical State University (Greensboro, NC, USA). The rearing and management of the chicks followed Aviagen’s (Huntsville, Al, USA) standard protocols for the Ross 708 strain [27]. Chicks were allocated to two treatments in a completely randomized design (CRD). The experimental treatments included a basal diet of corn-soybean meal (SBM) with a 3% dietary inclusion of one of two dietary fats: poultry fat (CON) or flaxseed oil (FLAX) (Table 1). The oil types used were procured commercially from Jedwards International, Inc. (Braintree, MA, USA). Experimental diets were calculated to be equicaloric and were manufactured at the North Carolina State University Feed Education Unit (Raleigh, NC, USA). Experimental diets were formulated to meet or slightly exceed the nutritional requirements outlined in the Ross broiler nutrition specification handbook [28] and fed to chicks as crumbled pellets throughout the 3-week experiment. Each treatment group was randomly assigned to 5 replicate pens (i.e., battery cages; Alternative Design Manufacturing and Supply Inc. (Siloam Springs, AR, USA), each containing 16 chicks. Each battery cage was fitted with a nipple drinker to supply water and a feeder tray, which was adjusted according to chick growth. The bird room temperature was 92 °F from d 1 to d 7, and 87 °F from d 8 to d 21. Photoperiod consisted of continuous (23L:1D) lighting from placement to 7 d, and 20L:4D from 8 d to 21 d [29]. Fatty acid profile of the Experimental Diets was listed in Supplementary Table S1.

### 2.2. Growth Performance Parameters

The performance parameters of the two treatments were routinely monitored during the 21-day experimental period. The chickens’ recorded body weights were used to analyze body weight gain (BWG) and determine the weight gained. Feed intake (FI), which shows the amount of feed consumed by the broiler chickens throughout the experiment, was also recorded daily and used to calculate the feed consumption. The feed conversion ratio (FCR), which is the measure of the efficiency with which broiler chickens convert feed into body weight, was measured. This was evaluated by dividing the total amount of feed consumed by the total weight gain.

### 2.3. Sample Collection

On d 21 of the experiment, a bird was randomly sampled from each pen. As described by Dennis et al. [30]. The dorsal raphe nucleus and hypothalamus were dissected using a stereotaxic atlas. The brain segments were placed in 2.0 mL cryogenic tube and then rapidly frozen in liquid Nitrogen (N_2_), and subsequently kept at −80 °C until further analyses to determine the influence of FLAX on the DRN and hypothalamus metabolite profiles via metabolomics. The blood sample was collected into blood capillary tubes, centrifuged, and the plasma was then transferred into microcentrifuge tubes and stored at −80 °C.

### 2.4. Sample Preparation for LC-MS

100 mg of each DRN and hypothalamus tissue sample was weighed into a prelabeled CK14 Precellys tube, thawed on ice separately, and homogenized (precellys 24 bertin technologies, Rockville, MD, USA) at 2500 rpm for 15 s. 1000 µL of 80% methanol extraction solvent was added to the homogenized samples, and the samples were re-homogenized. The subsequent liquid was transferred into prelabeled microcentrifuge tubes. Samples were centrifuged (Sorvall Legend Micro 21, Thermo Scientific, Waltham, MA, USA) for 8 min at 10,000 rpm. The supernatant was transferred into a new prelabeled microcentrifuge tube, and the pellet was discarded. The supernatant (solvent) was evaporated overnight in a Speedvac (SAVANT SPD2010, Thermo Scientific, Waltham, MA USA) concentrator. The dried extract was reconstituted for HPLC analysis by adding 75 µL of 95% water, 5% acetonitrile, 0.1% formic acid. The tubes were sonicated for 5 min and centrifuged for 8 min at 13,000 rpm. The supernatants were transferred to a Waters plastic HPLC vial (Waters Corporation, Milford, MA 01757, USA), and the pellet was discarded. Pooled quality control (QC) samples were prepared by combining aliquots from all the samples and were analyzed periodically during the analytic run to monitor the system’s performance.

#### 2.4.1. Quantification of Serotonin and 5-HIAA

An Agilent 1290 Infinity II liquid chromatography (LC) system coupled to an Agilent 6470 series QQQ mass spectrometer (MS/MS) was used to analyze samples. (Agilent Technologies, Santa Clara, CA, USA). An Acquity UPLC BEH Amide 2.1 mm × 100 mm, 1.7 µm column was used for LC separation (Waters Corp., Milford, MA, USA). The buffers were acetonitrile + 0.3% formic acid and acetonitrile/100 mM ammonium formate (20/80 *v*/*v*). The linear LC gradient was as follows: time 0 min, 0% B; time 1 min, 0% B; time 10 min, 50% B; time 11 min, 100% B; time 11.5 min, 0% B; time 15 min, 0% B. The flow rate was 0.3 mL/min, and the column was heated to 30 °C. Multiple reaction monitoring was used for MS analysis. Data were acquired in positive electrospray ionization (ESI) mode. The jet stream ESI interface had a gas temperature of 325 °C, gas flow rate of 8 L/minute, nebulizer pressure of 40 psi, sheath gas temperature of 250 °C, sheath gas flow rate of 7 L/minute, capillary voltage of 4000 V in positive mode, and nozzle voltage of 1500 V. The ΔEMV voltage was 500 V. Agilent Masshunter Quantitative analysis software was used for data analysis (version 10.1).

#### 2.4.2. LC-MS Analysis of Metabolite

All samples were acquired by the LC-MS system following the instructions of the instrument. An Agilent 1290 Infinity II liquid chromatography (LC) system coupled to an Agilent 6546 series QQQ mass spectrometer (MS/MS) was used for chromatographic separation (Agilent Technologies, Santa Clara, CA, USA). An Atlantis T3 2.1 mm × 75 mm, 3.0 μm column was used for LC separation (Waters Corp., Milford, MA, USA). The buffers were (A) water + 0.1% formic acid and (B) acetonitrile + 0.1% formic acid. The linear LC gradient was as follows: time 0 min, 5% B; time 0.5 min, 5% B; time 12 min, 70% B; time 13 min, 98% B; time 14 min, 5% B; time 20 min, 5% B. The flow rate was 0.3 mL/min, and the column was heated to 30 °C. Multiple reaction monitoring was used for MS analysis. Data were acquired in positive electrospray ionization (ESI) mode. The jet stream ESI interface had a gas temperature of 325 °C, gas flow rate of 10 L/min, nebulizer pressure of 40 psi, sheath gas temperature of 250 °C, sheath gas flow rate of 7 L/min, capillary voltage of 4000 V in positive mode, and nozzle voltage of 1500 V. The ΔEMV voltage was 400 V. The injection volume for each sample was 4 μL. A high-resolution Agilent mass spectrometer TripleTOF 6546 LC/Q-TOF (Agilent Technologies, Santa Clara, CA, USA) was used to detect metabolites eluted from the column. The Q-TOF was operated in both positive and negative ion modes. The curtain gas was set at 30 PSI, the ion source gas1 was set at 60 PSI, the ion source gas2 was set at 60 PSI, and the interface heater temperature was 650 °C. In the positive ion mode, the ion spray voltage floating was set at 5000 V. In the negative ion mode, the ions pray voltage floating was set at −4500 V. The mass spectrometry data were acquired in IDA mode. The TOF mass range was from 60 to 1200 Da. The survey scans were acquired in 150 ms, and as many as 12 product ion scans were collected if a threshold of 100 counts per second (counts/s) was exceeded and a 1+ charge-state was detected. The total cycle time was fixed to 0.56 s. Four times bins were summed for each scan at a pulse frequency value of 11 kHz through monitoring of the 40-GHz multichannel TDC detector with four-anode/channel detection. Dynamic exclusion was set at 4 s. During the acquisition, the mass accuracy was calibrated every 20 samples. Furthermore, to evaluate the stability of the LC-MS during the whole acquisition, a quality control sample (pool of all samples) was acquired after every 10 samples.

#### 2.4.3. Metabolite Data Processing

The acquired MS data pretreatments, including peak detection, peak grouping, alignment and gap filling for *m*/*z* values, retention time correction, second peak grouping, and annotation of isotopes and adducts, were performed using MS-DIAL software 4.9. LC-MS raw data files were converted into mzXML format, then processed by MS-DIAL and analyzed with the R version 4.4.1 software [31,32]. The datasets were normalized before analysis. Each ion was identified by combining retention time (RT) and *m*/*z* data. Intensities of each peak were recorded, and a matrix containing arbitrarily assigned peak indices (retention time-*m*/*z* pairs), sample names (observations), and ion intensity information (variables) was generated. The intensity of peak data was further pre-processed by MS-DIAL 4.9. Those features detected in less than 60% of QC samples were removed, and the remaining peaks with missing values were imputed with the k-nearest neighbor algorithm to improve the data quality further. PCA was performed for outlier detection and batch effects evaluation using the pre-processed dataset. A quality control-based signal correction was fitted to the QC data with respect to the order of injection to minimize signal intensity drift over time. For MS1 identification, the adducts [M+H]^+^, [M+Na]^+^, [2M+H]^+^, and [2M+Na]^+^ were used in the positive mode, with 10 ppm mass tolerance. For MS2 annotation, mass tolerance was set at 0.05 Da with an identification score cut-off of 75%. Online open-source KEGG and HMDB databases were used to annotate the metabolites by matching the exact molecular mass data (*m*/*z*) of samples with those from the database. If a mass difference between the observed and the database value was less than 10 ppm, the metabolite would be annotated, and the molecular formula of the metabolites would further be identified and validated by the isotopic distribution measurements.

### 2.5. Statistical Analysis

Data collected for variables BWG, FI, and FCR were subjected to a *t*-test in SAS (Statistical Analysis Software, 2004, Version 9.2. SAS Institute Inc., Cary, NC, USA). Similarly, serotonin and 5-HIAA concentrations were also analyzed using a *t*-test in SAS. Principal components analysis (PCA) was used to reduce data dimensionality and visualize sample clustering and separations based on the metabolite profiles [33,34]. Distinct metabolic pathways in the plasma and brain segments—KEGG pathway database, *Gallus gallus* library (KEGG) [35], and MetaboAnalyst 6.0 [36] to identify metabolic pathway profile changes in the plasma and brain segments. A two-way ANOVA (treatment × tissue) using conditional contrasts was used to identify significantly different metabolites between treatments within tissues [33], and the statistical analysis software used was R [32]. All means are presented as the Mean ± SD, and statistical significance was declared at *p* = 0.05.

## 3. Results

### 3.1. Growth Performance

After three weeks of the experimental trial, the BWG was significantly (*p* < 0.05) higher in FLAX compared to the CON. Although the FI between the FLAX and CON was not statistically (*p* < 0.05) different, FLAX had a significantly (*p* < 0.05) better FCR compared to CON. The dietary effect of FLAX on the growth performance of broiler chicks is presented in Table 2.

### 3.2. Serotonin and 5-HIAA Levels in the DRN and HYP

Serotonin and its primary metabolite 5-hydroxyindoleacetic acid (5-HIAA) were measured in the DRN and HYP of broiler chickens fed control or flaxseed oil FLAX diets (Table 3). In the DRN, serotonin levels were significantly higher in the FLAX group compared to CON. Similarly, in the HYP, serotonin level was increased in FLAX-fed birds relative to CON. In contrast, 5-HIAA levels did not differ between treatments in either the DRN or HYP. These results indicate that dietary flaxseed oil increased serotonin content in both serotonergic brain regions without significantly altering its major metabolite.

### 3.3. Metabolites Crossing Blood-Brain Barriers

**Figure 1:**
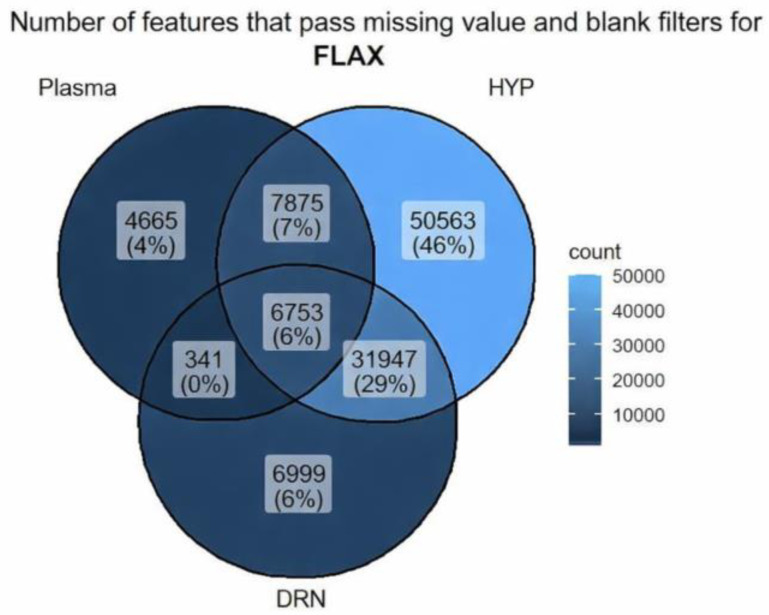
Number of features that pass missing values and blank filters across Plasma, DRN, and HYP.

The Venn diagram illustrates the number of features that pass missing values and blank filters across Plasma, DRN, and HYP for flax, as shown in Figure 1. HYP has the highest unique number of features, 50,563 (46%) 6999 (6%) and 4665 (4%) features in DRN and Plasma, respectively. This revealed that DRN and Plasma shared most of their features with each other or with HYP. Meanwhile, HYP exhibits many distinct features not found in the other two tissues.

**Figure 2:**
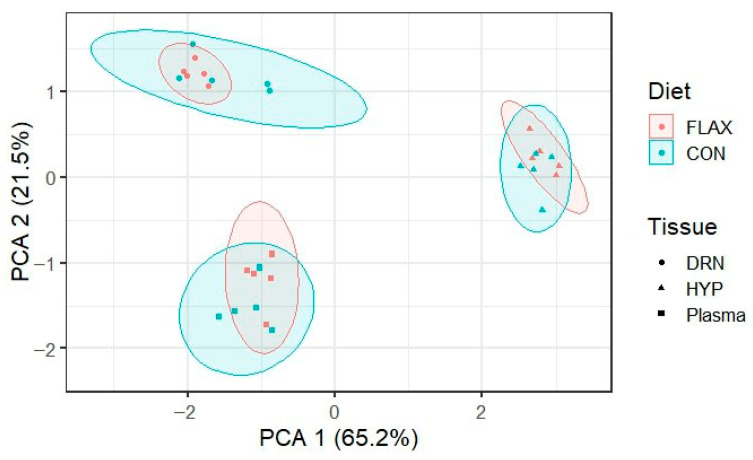
PCA showing the distribution of metabolites.

PCA was performed to visualize the distribution of metabolite profiles across tissue types (DRN, HYP, and Plasma) and dietary treatments (CON vs. FLAX). The score plot (Figure 2) shows that differences among tissues were substantially greater than differences between treatments within each tissue. PC1 (65.2%) and PC2 (21.5%) together explain 86.7% of the total variance, with clear separation observed between DRN, HYP, and plasma clusters. Within each tissue cluster, samples from CON and FLAX treatments completely overlap, indicating that dietary flaxseed oil did not induce many shifts in the overall metabolomic profile of the tissues.

Differentially abundant metabolites (*p* < 0.05) in the plasma, DRN, and HYP were identified using ANOVA, while KEGG and HMDB databases were used to annotate the metabolites. Results generated 2271 metabolites, of which 650 (*p* < 0.05) were annotated. Out of the 35 differentially expressed metabolites in the plasma, all eight (8) were differentially expressed in the brain, and all the 8 were upregulated in plasma, suggesting they may be capable of crossing the brain. The likely pathways and functional roles are shown in Table 4.

**Figure 3:**
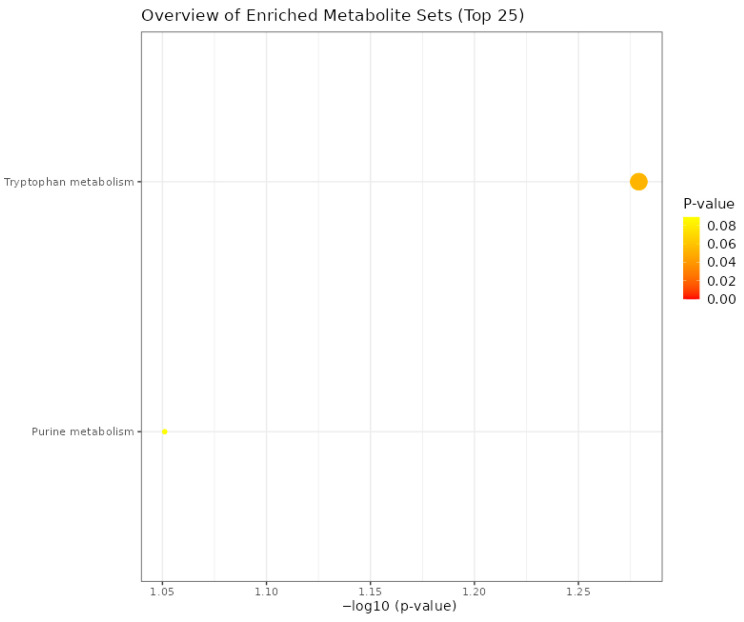
Dot plot overview of enriched metabolites.

All eight metabolites that possibly crossed the blood-brain barrier were uploaded to MetaboAnalyst 6.0 [36] for an enrichment overview. The dot plot of enriched metabolites revealed that tryptophan had the highest enrichment ratio compared to the purine metabolism (Figure 3).

## 4. Discussion

The type of fat in the diet plays a significant role in the growth performance of broiler chickens [37]. There are different reports on the impact of dietary fat types on the growth performance of broiler chicken studies, but the reported impacts differ among studies. In some studies, the dietary fat type did not significantly influence the growth performance parameters of chickens, especially the body weight gain and feed conversion ratio [37,38,39]. However, Huo et al. [40] reported that the degree of saturation of fats significantly influenced the growth performance of chickens. In this study, broilers fed dietary flaxseed oil had better BWG and FCR than those in the control group. Flaxseed oil contains polyunsaturated fatty acids, and the degree of saturation and fatty acid composition of fats has been reported to influence the digestibility of dietary fats [40]. This resulted in better digestibility, leading to better feed efficiency [37,40] as observed in the current study.

The principal component analysis revealed that the metabolomic profiles of the dorsal raphe nucleus, hypothalamus, and plasma clustered distinctly by tissue type, but samples from the control and flaxseed oil treatments overlapped completely within each tissue. This observation indicates that, despite the significant effects of flaxseed oil on individual metabolites, the overall global metabolomic signature of each tissue remained largely unchanged. This is not uncommon in nutritional metabolomics studies, where dietary interventions often alter specific metabolic pathways without shifting the entire metabolome [18,41]. The strong tissue separation reflects a fundamental difference in the metabolic functions among the DRN, HYP, and plasma, as expected given their distinct physiological roles. The absence of treatment-based clustering suggests that flaxseed oil exerts targeted, pathway-specific effects rather than a broad, nonspecific metabolic reprogramming. This reinforces the value of focused differential expression analysis for detecting subtle but biologically meaningful changes that might otherwise be obscured by dominant tissue-to-tissue variation [42,43].

The blood-brain barrier (BBB) maintains nutrients and ions at necessary levels for neuronal function and keeps neurotoxic substances away from the brain [44]. It is a strong barrier between the blood and brain parenchyma, giving a stable microenvironment that is crucial for intricate neural function and protecting the central nervous system (CNS) from chemical injury and damage [45]. The BBB protects the brain by limiting the movement of substances into it. It also plays a vital role in supplying essential nutrients and eliminating metabolic waste [46]. For some of these metabolites to be available and exert their biological properties in the serotonergic region of the brain, the BBB must be crossed. However, detecting a metabolite in both plasma and brain does not confirm trans BBB transport, it could also result from brain synthesis or other uptake mechanisms. Compounds can cross the BBB through passive transport, either transcellularly via the phospholipid bilayer of the membranes of the BBB endothelial cells or paracellularly between endothelial cells, where tight junctions restrict molecule diffusion. The effectiveness of diffusion is dependent on physiochemical properties such as lipid solubility and molecular weight of the compounds [47]. Although the presence of flavonoids in plasma does not guarantee their entry into the central nervous system, it is a necessary first step, as circulating compounds must traverse the blood-brain barrier to exert direct effects on brain tissue [48]. In this study, a total of 8 metabolites were found to be differentially expressed in both the plasma and the brain.

Corydaline and galanthamine are naturally occurring alkaloids with reported neuroprotective properties in mammalian models. Both compounds have been reported to cross the BBB due to their lipophilic properties [49,50]. In this study, corydaline was upregulated in the DRN and galanthamine in the HYP of FLAX-fed birds. These natural compounds have been established to markedly reduce the deterioration of neuronal cells caused by oxidative stress-mediated neurotoxicity [51]. Galanthamine is a known inhibitor of acetylcholinesterase (AChE), an enzyme that breaks down the neurotransmitter acetylcholine [52]. By inhibiting AChE, galanthamine could potentially prolong the action of acetylcholine in the brain. The upregulation of these compounds suggests a potential role of FLAX in enhancing chickens’ neuroprotection and modulate cholinergic neurotransmission.

Xanthoxyletin, an anti-inflammatory compound, was downregulated in the HYP, while gambogic acid, an antioxidative compound, was upregulated. Xanthoxyletin is a naturally occurring compound that belongs to the family of coumarins, found in plants like citrus and Zanthoxylum. Coumarins are plant-derived compounds that have attracted intense interest recently due to their diverse and potent pharmacological properties [44,53]. Their effects on the central nervous system (CNS) have been established [44]. An in vitro study conducted by Yang et al. [44] revealed that coumarin derivative compounds cross the BBB and exert anti-inflammatory properties. The detection of xanthoxyletin in both plasma and HYP in this study is consistent with possible permeability. Neuroinflammation is a significant contributor to neurodegenerative diseases, which could impair animals’ cognitive function and behavior [54]. Coumarin derivatives have shown the ability to reduce neuroinflammation in human and mouse cells [55] and also inhibit enzymes such as cyclooxygenase (COX) and lipoxygenase (LOX) involved in the production of inflammatory mediators [55]. The resultant inhibition outcome dampens the inflammatory response. This highlights their therapeutic potential in neuroinflammatory diseases [55]. Meanwhile, the downregulation of xanthoxyletin, an anti-inflammatory compound in this study, could be attributed to polyunsaturated fatty acids in FLAX, which improves the broiler’s brain health.

Mitochondria are the powerhouse of animals, responsible for generating nearly all the chemical energy required to power the cell’s biochemical reactions. Despite making up a small percentage (2%) of the total body weight of animals, the brain consumes about 20% of the body’s total energy. Mitochondria usually produce reactive oxygen species (ROS) as a byproduct of energy production. The ROS generated can be regulated by non-enzymatic antioxidants, such as glutathione, and antioxidant enzymes, such as superoxide dismutase [56,57]. The balance between ROS produced and antioxidants is very important, as ROS accumulation can cause oxidative damage to brain biomolecules [57]. The brain is energy-demanding and relies heavily on mitochondria to meet its metabolic needs. It has been reported that there is a relationship between mitochondrial dysfunction and behavioral changes, which is due to impairment in energy and high levels of oxidative stress [58]. Therefore, compounds that enhance antioxidant defenses or mitochondrial function in the brain are important for healthy behavior. Yang [57] reported that gambogic acid is an important antioxidant capable of reducing ROS and activating cell apoptosis. The upregulation of gambogic acid in the HYP in this present study suggests the antioxidant capacity of the FLAX.

Dethiobiotin and xanthosine 5′-monophosphate are not expected to cross the blood brain barrier via passive diffusion due to their polarity. However, both were upregulated in brain tissues of flaxseed oil fed birds. This suggests that dietary flaxseed oil may influence their local synthesis or metabolism within the central nervous system, rather than their import from plasma. Dethiobiotin is a precursor in biotin synthesis and is actively transported into the brain via the sodium-dependent multivitamin transporter (SMVT). Its deficiency in chickens has been linked to congenital malformations [59]. An increase in the concentration level of biotin in the brain region’s hippocampal tissues is crucial for learning and memory [60,61], while also playing a significant role in energy production as it acts as a co-factor for enzymes to help convert carbohydrates, proteins, and fats into usable energy. Flaxseed oil could potentially upregulate SMVT expression or activity, leading to increased biotin related intermediates in the brain. Similarly, xanthosine 5-monophosphate acts as an intermediate in the purine metabolism pathway which leads to the generation of nucleotides such as adenosine triphosphate (ATP). The tryptophan hydroxylase, the rate-limiting enzyme in serotonin biosynthesis, is ATP-dependent. Since ATP provides the required energy for synaptic vesicle transport, exocytosis, and receptor activation during serotonin-mediated neurotransmission [62], the upregulation of dethiobiotin and xanthosine 5-monophosphate in this study suggests that flaxseed oil may be associated with increased energy availability in the brain, which could hypothetically contribute to behavioral regulation. This enhanced brain energy metabolism could support improved neural efficiency and systemic growth, aligning with the observed better feed conversion ratio and body weight gain in FLAX-fed birds. Thus, the upregulation of these energy-related metabolites may represent a mechanistic link between dietary flaxseed oil, central energy status, and peripheral growth performance.

The enrichment overview of the metabolites from this study revealed tryptophan metabolism as the most enriched metabolic pathway. Tryptophan is a major substrate for the synthesis of serotonin (5-hydroxytryptamine, 5-HT) in the brain, and the DRN is the main source of serotonergic neurons. The DRN in broiler chickens forms a significant part of the serotonergic system and helps regulate physiological and behavioral functions [29] and remains a vital structure in the brainstem and plays a significant role in maintaining and carrying out brain functions, such as cognitive processes, neuroendocrine functions, and synaptic plasticity [20,21]. Dietary fat types can impact the neural balances that release neurotransmitters like serotonin, influencing metabolic functions and bird behavior. In the DRN, neurotransmission embodies a complex interplay of processes involving synthesis, release, activation of receptors, reuptake, and degradation back into the neural cells. Lizarbe [22] reported that the amount and composition of dietary fats have been linked to impaired functions and modifications of the brain. In serotonergic neurons, tryptophan is the major precursor for serotonin (5-hydroxytryptamine, 5-HT). The metabolic pathway of 5-HT is initiated by tryptophan, which is hydroxylated to the intermediate 5-hydroxytryptophan (5-HTP), which is then decarboxylated to become 5-HT [63,64]. In animals, different studies have suggested that the neuromodulatory functions of serotonin are connected with a broad range of processes, including behavior, cognition, and emotion [29,65,66]. In recent years, one of the main goals of the poultry industry has been to ensure that, while improving broiler chicken performance, the overall health and welfare of the birds are maintained [29]. The poor welfare of production animals can be revealed by measuring the affective state of the animals. Poorer welfare is linked to negative affective states such as fearfulness [67]. Lundgren [67] reported that the manipulation of the serotonin pathway through dietary manipulation of 5-hydroxytryptophan significantly reduced measured fearfulness in red jungle fowl. There is a significant relationship between the polyunsaturated fatty acids and tryptophan metabolism, as a study has shown that omega-3 polyunsaturated fatty acids supplementation, especially eicosapentaenoic acid (EPA) and docosahexaenoic acid (DHA), improves tryptophan metabolism [68]. Flaxseed oil is rich in EPA and DHA, which could possibly explain the reason for the impact of the tryptophan metabolism pathway in this study.

Serotonin synthesis in serotonergic neurons is limited by the availability of its precursor, tryptophan [69]. In this study, dietary flaxseed oil significantly elevated serotonin levels in both the dorsal raphe nucleus and hypothalamus. This increase is consistent with the availability of tryptophan observed in the DRN, which likely enhanced flux through the serotonin synthesis pathway. These findings align with the established principle that brain tryptophan levels are a key determinant of serotonin formation [70]. The dorsal raphe nucleus is the primary source of serotonergic projections throughout the brain and is involved in the regulation of mood and stress responses, while the hypothalamus acts as a central regulator of homeostasis and is similarly modulated by serotonergic inputs [71,72,73]. The elevation of serotonin within these regions, therefore, suggests that dietary flaxseed oil may influence physiological processes such as behavior and stress-related responses [74,75]. Despite the significant increase in serotonin, 5-hydroxyindoleacetic acid (5-HIAA) levels did not differ significantly between treatments in either the dorsal raphe nucleus or hypothalamus. This suggests that the elevated serotonin is not a consequence of increased catabolism, but rather reflects increased synthesis or altered vesicular storage and release [76].

FLAX contains omega-3 fatty acids, ALA, with reported health benefits in other species. In this study, we observed metabolite changes that may be consistent with anti-inflammatory activity. ALA can be converted to DHA and EPA, which have been reported to have health benefits against neurological disorders and immunological diseases [77,78]. Various studies have shown that polyunsaturated fatty acids from FLAX have great anti-inflammatory activity [68]. However, with the nutritional benefits of FLAX, they contain high levels of phytotoxic compounds. The consumption of these phytotoxic compounds can have a negative effect on animals. Flaxseed is reported to contain 394.99 mg/kg secondary metabolites of plants known as cyanogenic glycosides, such as lotaustralin [79,80]. In the intestine, the glycosides can be converted to cyanohydrin by intestinal β-glycosidase and then decomposed to hydrogen cyanide [81]. Acute cyanide poisoning from hydrogen cyanide may threaten neurological and respiratory systems [79]. In this study, lotaustralin was upregulated in the HYP, although the way in which it crosses the BBB is unknown. Nevertheless, no clinical signs of toxicity were observed in the FLAX-fed birds, suggesting that 3% inclusion level of flaxseed oil was safe under the conditions of this study. However, function significance of lotaustralin accumulation in the brain needs further study.

## 5. Conclusions

This study showed dietary flaxseed oil’s significant impact on metabolites crossing broilers’ blood-brain barrier. The results showed that polyunsaturated fatty acids (PUFAs) from flaxseed oil can potentially modify brain chemistry by modulating major metabolites. The upregulation of metabolites such as tryptophan, dethiobiotin, and xanthosine 5′-monophosphate suggests potential benefits of flaxseed oil in neuroprotection, energy metabolism, and serotonin regulation. Meanwhile, the upregulation of the cyanogenic glycoside lotaustralin raises concerns about potential toxicity, necessitating further investigation into its effects on brain health. The findings from this study provide insights into the modulation of HYP and DRN metabolite profile in broiler chicks given flaxseed oil-based diet. However, further study is required to fully understand the mechanisms by which some of these metabolites influence the chick’s behavior and health. 

## Figures and Tables

**Table 1:** Composition of experimental starter diets (%) ^1^.

Ingredients	CON	FLAX
Corn	53.22	53.22
Soybean Meal	39.40	39.40
Fat/Oil *	3.00	3.00
Mono-Dicalcium Phosphate	1.81	1.81
Limestone 37%	0.95	0.95
Salt NaCl	0.45	0.45
DL-Methionine	0.35	0.35
NCSU Poultry Mineral Premix ^2^	0.20	0.20
Choline Chloride 60%	0.20	0.20
L-Lysine	0.18	0.18
L-Threonine	0.09	0.09
NCSU Poultry Vitamin Premix ^3^	0.05	0.05
Selenium Premix ^+^	0.05	0.05
Santoquin	0.05	0.05
**Analyzed nutrient composition**		
Metabolizable Energy (Kcal/kg)	3152.6	3143.8
Crude Protein, %	23.06	23.19
Crude Fat, %	5.42	5.12
Crude Fiber, %	2.1	2.3
Ash, %	5.64	5.55
**Calculated nutrient composition**		
Total Sulfur Amino Acids, %	0.19	0.19
Lysine, %	1.44	1.44
Calcium, %	0.96	0.96
Available phosphorus, %	0.48	0.48

^1^ Diets used in the study included the following: (i) conventional Corn-soybean meal (SBM) with the addition of poultry fat as fat type (CON diet); (ii) conventional corn-SBM with flaxseed oil as fat type (FLAX). * The two different fat types were added at 3% in each diet. ^2^ Mineral Premix, supplied per kilogram of diet: Manganese (Mn), 60 mg; Zinc (Zn), 60 mg; Iron (Fe), 40 mg; Copper (Cu), 5 mg; Iodine (I), 1.2 mg; Cobalt (Co), 0.5 mg. ^3^ Vitamin Premix, supplied per kilogram of diet: Vitamin A (6600 IU), Vitamin D (1980 IU), Vitamin E (33 IU), Vitamin B12 (0.02 mg), Biotin (0.13 mg), Menadione (1.98 mg), Thiamine (1.98 mg), Riboflavin (6.60 mg), d-Pantothenic Acid (11.0 mg), Vitamin B6 (3.96 mg), Niacin (55.0 mg), Folic Acid (1.1 mg). Experimental diets were analyzed for proximate nutrient composition by Eurofins Scientific Inc. Nutrient Analysis Center, 2200 Rittenhouse Street, Suite 150, Des Moines, IA 50321, USA. ^+^ Selenium Premix provides 0.3 mg Selenium/Kg of feed as sodium selenite.

**Table 2:** Dietary effect of FLAX on growth performance of broiler chicks.

Treatments	BWG (Kg/Bird)	FI (Kg/Bird)	FCR (Kg:Kg)
CON	0.8132 ± 0.02105 ^b^	0.09661 ± 0.02243	1.1882 ± 0.01230 ^a^
FLAX	0.8725 ± 0.02817 ^a^	1.0106 ± 0.04144	1.1582 ± 0.01233 ^b^
*p*-value	0.0055	0.0674	0.0049

^a,b^ Mean value bearing different superscript letters within a column are significantly different (*p* < 0.05). BWG—Body Weight Gain, FI—Feed Intake, and FCR—Feed Conversion Ratio.

**Table 3:** Serotonin and 5-HIAA concentrations in the HYP and DRN.

Treatments	Serotonin HYP	Serotonin DRN	5-HIAA HYP	5-HIAA DRN
CON	0.0048 ± 0.00350 ^b^	0.0015 ± 0.00019 ^b^	0.2838 ± 0.0256	0.2952 ± 0.0194
FLAX	0.0292 ± 0.0423 ^a^	0.0070 ± 0.00556 ^a^	0.2501 ± 0.0384	0.2232 ± 0.0191
*p*-value	0.0136	0.0022	0.6146	0.9833

^a, b^ Mean value bearing different superscript letters within a column are significantly different (*p* < 0.05). HYP—Hypothalamus, DRN—dorsal raphe nucleus, and 5-HIAA—5-hydroxyindoleacetic acid. Units: ng/mg tissue.

**Table 4:** Metabolites detected in both plasma and brain tissues.

Metabolite Name	Tissue	Regulation	*p*-Value	Pathway	Functional Roles
Tryptophan	DRN	Up	0.0018	Serotonergic pathway	Mood, Cognition
	HYP	-	0.6737		
	Plasma	Up	0.0022		
Xanthosine 5′-monophosphate	DRN	Up	0.0353	Serotonin synthesis	Serotonin signaling
	HYP	-	0.6266		
	Plasma	Up	0.0010		
	DRN	Up	0.0448	NF-κB Signaling pathway	Inflammation & immune response
Corydaline	HYP	-	0.4305		
	Plasma	Up	0.0029		
	DRN	-	0.1795	Serotonin synthesis	Serotonin signaling
Dethiobiotin	HYP	Up	0.0032		
	Plasma	Up	0.0001		
	DRN	-	0.9778	ROS production pathway	Oxidative stress
Lotaustralin	HYP	Up	0.0080		
	Plasma	Up	0.0013		
	DRN	-	0.5380	NF-κB Pathway Inhibition	Anti-inflammatory
Xanthoxyletin	HYP	Dn	0.0311		
	Plasma	Up	0.0211		
	DRN	-	0.7364	Cholinergic Neurotransmission Pathway	Cognitive function
Galanthamine	HYP	Up	0.0027		
	Plasma	Up	0.0022		
Gambogic acid	DRN	-	0.1699	ROS modulation	Neuronal protection
	HYP	Up	0.0058		
	Plasma	Up	0.0012		

Up—Upregulated, Dn—Downregulated, and ROS—Reactive Oxygen Species.

## Data Availability

The original contributions presented in this study are included in the article/Supplementary Material. Further inquiries can be directed to the corresponding author.

## Supplementary Materials

The following supporting information can be downloaded at: https://www.mdpi.com/article/10.3390/biom16050661/s1, Supplementary Table S1: Fatty acid composition of experimental diets.
