# Supplementary material for: Modulating Blood-Brain Barrier Metabolites of Broiler Chickens Through Dietary Flaxseed Oil
Source: Biomolecules. 2026 Apr 29;16(5):661. doi: 10.3390/biom16050661 (PMC13204195; doi:10.3390/biom16050661)
Supplement: Supplementary file 1 [file biomolecules-16-00661-s001.zip › biomolecules-4220273-supplementary.pdf]

Supplementary Table S1: Fatty acid composition of experimental diets[1]

Starter Diets		
**Fatty acids[2]**	**CON (%)**	**FLAX (%)**
C4:0	< 0.02	< 0.02
C6:0	< 0.02	< 0.02
C8:0	< 0.02	< 0.02
C10:0	< 0.02	< 0.02
C11:0	< 0.02	< 0.02
C12:0	< 0.02	< 0.02
C14:0	0.02	0.03
C14:1	< 0.02	< 0.02
C15:0	< 0.02	< 0.02
C15:1	< 0.02	< 0.02
C16:0	1.12	0.6
C16:1	0.19	< 0.04
C16:2	< 0.02	< 0.02
C16:3	< 0.02	< 0.02
C16:4	< 0.02	< 0.02
C17:0	< 0.02	< 0.02
C17:1	< 0.02	< 0.02
C18:0	0.25	0.17
C18:1	1.75	1.21
C18:2	1.95	1.75
C18:3 n-3	0.12	1.1
C18:3 n-6	< 0.02	< 0.02
C18:4	< 0.02	< 0.02
C20:0	< 0.02	< 0.02
C20:1	< 0.02	< 0.02
C20:2	< 0.02	< 0.02
C20:3 n-3	< 0.02	< 0.02
C20:3 n-6	< 0.02	< 0.02
C20:4 n-3	< 0.02	< 0.02
C20:4 n-6	< 0.02	< 0.02
C20:5 n-3	< 0.02	0.05
C21:5	< 0.02	< 0.02
C22:0	< 0.02	< 0.02
C22:1	< 0.02	< 0.02
C22:2	< 0.02	< 0.02
C22:3	< 0.02	< 0.02
C22:4	< 0.02	< 0.02
C22:5 n-3	< 0.02	< 0.02
C22:5 n-6	< 0.02	< 0.02

C22:6	< 0.02	0.03
C24:0	< 0.02	< 0.02
C24:1	< 0.02	< 0.02
Total n3	0.12	1.2
Total n5	< 0.05	< 0.05
Total n6	1.99	1.74
Total n7	0.27	0.09
Total n9	1.77	1.23
Total fatty acids	5.67	5.18
MUFAs	2.07	1.35
PUFAs	2.13	2.97
SFAs	1.44	0.85
Metabolizable Energy (Kcal/kg)	3,152.6	3,143.8
Crude Protein, %	23.06	23.19
Crude Fat, %	5.42	5.12
Crude Fiber, %	2.1	2.3
Ash, %	5.64	5.55

[1]Experimental diets were analyzed for fatty acid composition by Eurofins Scientific Inc. Nutrient Analysis Center, 2200 Rittenhouse Street, Suite 150, Des Moines, IA 50321.

[2]SFA =Saturated fatty acids; MUFA = monounsaturated fatty acids; PUFA = polyunsaturated fatty acids.
